# Immunological and Inflammatory Biomarkers in the Prognosis, Prevention, and Treatment of Ischemic Stroke: A Review of a Decade of Advancement

**DOI:** 10.3390/ijms26167928

**Published:** 2025-08-16

**Authors:** Marius P. Iordache, Anca Buliman, Carmen Costea-Firan, Teodor Claudiu Ion Gligore, Ioana Simona Cazacu, Marius Stoian, Doroteea Teoibaș-Şerban, Corneliu-Dan Blendea, Mirela Gabriela-Irina Protosevici, Cristiana Tanase, Maria-Linda Popa

**Affiliations:** 1 Faculty of Medicine, “Titu Maiorescu” University, 040441 Bucharest, Romania; buliman_anca@yahoo.com (A.B.); doroteeateoibas@yahoo.com (D.T.-Ş.); danblendea@gmail.com (C.-D.B.); irina.mirela.protosevici@gmail.com (M.G.-I.P.); 2 Ilfov County Clinical Emergency Hospital, 022104 Bucharest, Romania; firancarmen@yahoo.com; 3 STB Health Centre, 022106 Bucharest, Romania; 4 National Institute of Medical Expertise and Work Capacity Recovery, 050659 Bucharest, Romania; drclaudiugligore@gmail.com; 5 Neuromuscular Rehabilitation Clinic Division, Clinical Emergency Hospital “Bagdasar-Arseni”, 041915 Bucharest, Romania; ioanas.cazacu@gmail.com; 6 Hermes Medical Clinic, 077167 Ilfov, Romania; marius.stoian@ymail.com; 7 “Nicolae Cajal” Medical Institute, Faculty of Medicine and Pharmacy, “Titu Maiorescu” University, 011413 Bucharest, Romania; 8 “Victor Babeș” National Institute of Pathology, 050096 Bucharest, Romania; 9 Department of Cell Biology and Histology, “Carol Davila” University of Medicine and Pharmacy, 050474 Bucharest, Romania; maria_lindabv@yahoo.com

**Keywords:** ischemic stroke, inflammation, cytokines, biomarkers, immune response, prognosis, prevention, outcome

## Abstract

Ischemic stroke triggers a dynamic immune response that influences both acute damage and long-term recovery. This review synthesizes a decade of evidence on immunological and inflammatory biomarkers in ischemic stroke, emphasizing their prognostic and therapeutic significance. Following ischemic insult, levels of pro-inflammatory cytokines, such as interleukin-1β (IL-1β), interleukin-6 (IL-6), and tumor necrosis factor-α (TNF-α), and chemokines like interleukin-8 (IL-8) rapidly rise, promoting blood–brain barrier disruption, leukocyte infiltration, and neuronal death. Conversely, anti-inflammatory mediators such as interleukin-10 (IL-10) and transforming growth factor-β (TGF-β) facilitate repair, neurogenesis, and immune regulation in later phases. The balance between these pathways determines outcomes and is reflected in circulating biomarkers. Composite hematological indices including the neutrophil-to-lymphocyte ratio (NLR), platelet-to-lymphocyte ratio (PLR), and systemic immune-inflammation index (SII) offer accessible and cost-effective prognostic tools. Several biomarkers correlate with infarct size, neurological deterioration, and mortality, and may predict complications like hemorrhagic transformation or infection. Therapeutic strategies targeting cytokines, especially IL-1 and IL-6, have shown promise in modulating inflammation and improving outcomes. Future directions include personalized immune profiling, real-time cytokine monitoring, and combining immunotherapy with neurorestorative approaches. By integrating immune biomarkers into stroke care, clinicians may enhance risk stratification, optimize treatment timing, and identify candidates for novel interventions. This review underscores inflammation’s dual role and evolving therapeutic and prognostic relevance in ischemic stroke.

## 1. Introduction

Ischemic stroke remains a global health burden and a leading cause of disability and death. While its acute management has advanced, particularly with the advent of mechanical thrombectomy and thrombolytics, long-term functional outcomes vary widely between patients. Traditional prognostic tools rely heavily on clinical scales and imaging, but in recent years, biomarkers reflecting systemic and neuroinflammation have gained substantial attention [1]. The immune system plays a dual role in stroke. On one hand, inflammation increases the blood–brain barrier (BBB) permeability, augments oxidative stress, and promotes neuronal injury [2,3,4]. On the other, immune-regulated processes also facilitate neurorepair, angiogenesis, and debris clearance in subacute and chronic phases. This duality positions inflammatory mediators not just as mechanistic components, but also as potential biomarkers and therapeutic targets [5,6].

Key cytokines such as IL-6, IL-1β, TNF-α, and chemokines like IL-8 are rapidly released after stroke onset and are strongly associated with infarct size, neurological deterioration, and poor functional recovery [7,8,9,10]. Meanwhile, levels of anti-inflammatory cytokines such as IL-10 and TGF-β rise in the resolution phase and are linked not only to tissue repair but also to stroke-induced immunosuppression and increased infection risk [11,12]. Composite biomarkers such as the NLR, PLR, and SII (=platelet count × neutrophil count/lymphocyte count) provide a cost-effective means of assessing immune balance and have shown predictive value for stroke severity and outcomes in large cohorts [13,14,15]. At the same time, immunogenetic variants and peripheral inflammatory signatures offer further granularity to individual prognosis. Inflammation is not only a consequence but also a modifiable driver of stroke outcomes. Trials with agents like IL-1 receptor antagonists (IL-1Ra), tocilizumab (IL-6R antibody), and colchicine (NLRP3 (NOD-like Receptor Family Pyrin Domain-Containing 3) inflammasome inhibitor) suggest that immune-targeted interventions could enhance neuroprotection or prevent recurrence [16,17].

Despite this progress, translation into routine clinical practice remains limited. The evidence is fragmented across cytokine families, outcome timepoints, and stroke subtypes [1,18,19]. Thus, this review synthesizes a decade of the literature on immunological and inflammatory biomarkers in ischemic stroke. Our objective is to identify and classify key biomarkers, examine their associations with clinical outcomes, and explore emerging therapeutic strategies grounded in immune modulation.

## 2. Inflammation and Immune Response in Ischemic Stroke

The brain’s immune privilege is challenged during stroke, as cell injury and necrosis release damage-associated molecular patterns (DAMPs) that activate innate immune pathways [20,21]. Within minutes of an ischemic insult, resident microglia become activated and begin producing pro-inflammatory cytokines such as IL-1β and TNF-α [22,23]. Microglial activation is an early and dual-edged event, initially beneficial in debris clearance and trophic support, but ultimately harmful due to production of inflammatory mediators and neurotoxic substances [23,24].

**Figure 1:**
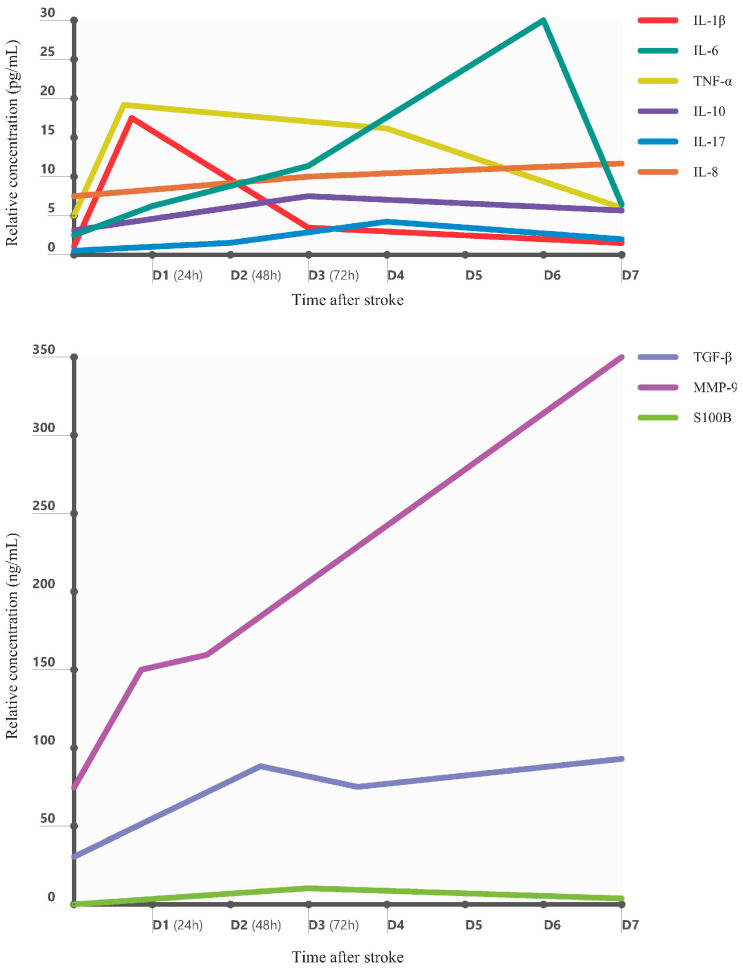
Temporal profile of relevant biomarkers post-stroke. Biomarkers shown (**top**): IL-1β (red): peaks early (D1), then rapidly declines by D3. IL-6 (green): rises steadily, peaks sharply at D6, then declines. TNF-α (yellow): rises early (D1), remains elevated until D4, then gradually declines. IL-10 (purple): shows a mild increase by D2 and a gradual decrease afterward. IL-17 (blue): increases modestly by D3 and then declines. IL-8 (orange): remains relatively stable with a slight upward trend. Biomarkers shown (**bottom**): TGF-β (blue): rises until D2, dips slightly at D3, then continues to rise by D7. MMP-9 (purple): sharp increase starting at D1, increases consistently through D7. S100B (green): small increase peaking at D3, then declines. Figure abbreviations: IL-1β (interleukin-1 beta), IL-6 (interleukin-6), TNF-α (tumor necrosis factor-alpha), IL-8 (interleukin-8), IL-10 (interleukin-10), IL-17 (interleukin-17), TGF-β (transforming growth factor-β), MMP-9 (matrix metalloproteinase-9), S100B (S100 calcium-binding protein B, used as a biomarker of astroglial activation or injury).

As the blood–brain barrier (BBB) becomes compromised, peripheral immune cells infiltrate the brain parenchyma. Neutrophils are among the earliest responders, reaching the infarct zone within hours and peaking around 24 h post-insult. They release matrix metalloproteinases (MMPs), including MMP-9, and reactive oxygen species (ROS), which exacerbate BBB breakdown and worsen neuronal damage [25,26]. Monocytes and lymphocytes follow in a temporally distinct pattern: monocyte-derived macrophages accumulate over days 3–7, while T lymphocytes infiltrate around day 2–3, secreting additional cytokines that propagate the inflammatory milieu. The sustained invasion of leukocytes contributes to secondary injury and neurological worsening [27,28,29]. Levels of IL-8 (CXCL8) and the acute-phase protein C-reactive protein (CRP) rise slightly later, with CRP peaking at approximately 48 h post-stroke [30,31]. In contrast, IL-10, a key anti-inflammatory cytokine, peaks between 24 and 72 h post-stroke as part of the immune system’s counter-regulatory response [30]. Interleukin-17 (IL-17), produced primarily by infiltrating T-helper 17 (Th17) and γδ T cells, shows a delayed elevation beginning around 48–72 h post-stroke, coinciding with later-stage T-cell infiltration and extended inflammation [32]. Figure 1 illustrates the time-course of key inflammatory mediators in the systemic circulation after an ischemic stroke.

**Figure 2:**
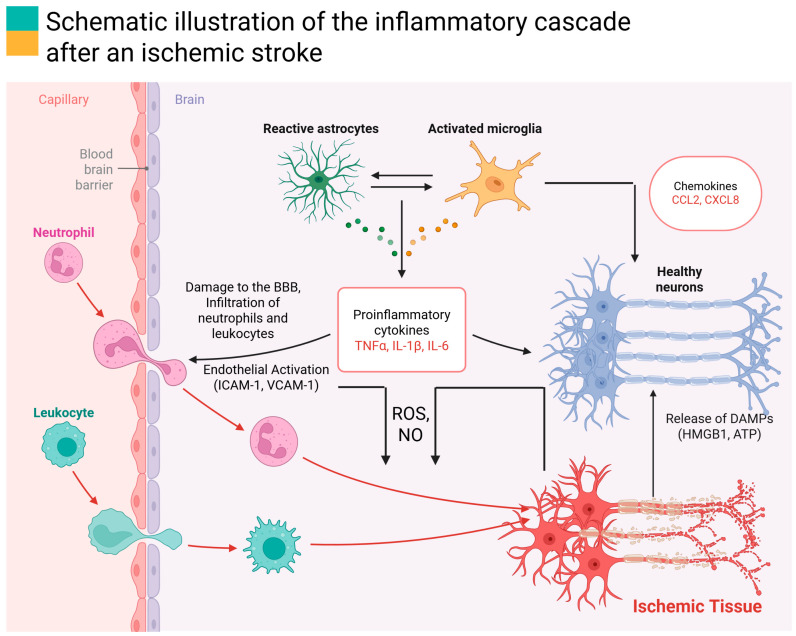
Schematic illustration of the inflammatory cascade after an ischemic stroke. Brain-blood barrier (BBB) disruption after cerebral ischemia, leading to the following: (i) infiltration of neutrophils and leukocytes into the brain parenchyma; (ii) endothelial activation, marked by increased expression of adhesion molecules (ICAM-1, VCAM-1); (iii) cellular response: infiltrated neutrophils and leukocytes release ROS and NO (that exacerbate tissue damage); (iv) glial activation: astrocytes become reactive, and microglia are activated and release (v) pro-inflammatory cytokines TNF-α, IL-1β, and IL-6; (vi) chemokines such as CCL2 and CXCL8 are released by glial cells, enhancing immune cell attraction; (vii) dying or stressed neurons release DAMPs (HMGB1 1, ATP) which further activate microglia and immune responses. Figure abbreviations: DAMPs (damage-associated molecular patterns), HMGB1 (High-Mobility Group Box 1), ATP (Adenosine Triphosphate), IL-1β (interleukin-1 beta), TNF-α (tumor necrosis factor-alpha), IL-6 (interleukin-6), CCL2 (C-C motif chemokine ligand 2, also historically known as MCP-1 (Monocyte Chemoattractant Protein-1)), CXCL8 (C-X-C motif chemokine ligand 8, more commonly known as interleukin-8), ICAM-1 (intercellular adhesion molecule-1), VCAM-1 (vascular cell adhesion molecule-1), ROS (reactive oxygen species), NO (Nitric Oxide).

Once recruited to the ischemic territory, immune cells engage in complex interactions. DAMPs such as HMGB1 (High-Mobility Group Box 1) and extracellular ATP (Adenosine Triphosphate) released from necrotic cells bind to pattern recognition receptors (e.g., TLRs (toll-like receptors), RAGE (Receptors for Advanced Glycation End-products), P2X7 (Purinergic Receptor P2X, Ligand-Gated Ion Channel, 7)) on microglia and other immune cells, amplifying inflammatory signaling and transmigration across the BBB [26,33,34]. This facilitates the orchestrated recruitment of immune cells and establishes a dynamic interplay between innate and adaptive immunity. While the infiltration of leukocytes can contribute to debris clearance and subsequent tissue repair, excessive or prolonged invasion aggravates inflammatory signaling and disruption of neurovascular integrity [35]. Pro-inflammatory cytokines such as IL-1β, IL-6, and TNF-α aggravate neuronal apoptosis via activation of death receptor pathways, destabilization of mitochondrial function, and exacerbation of excitotoxicity, which further promotes immune cell recruitment [9,22,36]. Figure 2 illustrates the key components of the inflammatory cascade after stroke, where an initial wave of cytokine release triggers a cycle of immune cell recruitment and further cytokine production, leading to expansion of tissue injury.

Neurons themselves, particularly in the ischemic penumbra, become an active source of cytokine release, amplifying tissue damage. IL-10, an anti-inflammatory cytokine, is upregulated as a compensatory response to counteract this pro-inflammatory surge but often fails to fully neutralize acute neurotoxicity if the inflammatory response is overwhelming [37,38]. Despite this, inflammation is not inherently deleterious; its role is highly phase dependent. In the subacute and chronic phases, microglia and macrophages can polarize toward an anti-inflammatory phenotype, secreting trophic factors and cytokines that support tissue remodeling and axonal sprouting. IL-10 and TGF-β are central to this reparative shift, promoting immune resolution and neurovascular stabilization. Notably, IL-1β, typically associated with acute injury, also contributes to later regenerative processes by stimulating angiogenesis and remodeling of endothelial cells during the repair phase [19,33].

As immune cells continue to accumulate, adhesion processes become central. Endothelial cells upregulate intercellular adhesion molecule-1 (ICAM-1), vascular cell adhesion molecule-1 (VCAM-1), and selectins in response to cytokine stimulation and hypoxia. These molecules interact with leukocyte integrins, including LFA-1 (Lymphocyte Function-Associated Antigen 1) (CD (Cluster of Differentiation) 11a/CD 18), Mac-1 (Macrophage-1 Antigen, also known as CD11b/CD18 or Integrin αMβ2), and VLA-4 (Very Late Antigen-4, also known as integrin α4β1 or CD49d/CD29), to mediate firm adhesion and transmigration into the ischemic tissue. The balance of T cell responses further shapes post-stroke neuroinflammation. Conversely, Th2 and Treg cells produce interleukin-4 (IL-4), interleukin-5 (IL-5), interleukin-13 (IL-13), IL-10, and TGF-β, contributing to immune suppression and repair. CD8^+^ cytotoxic T cells can directly induce neuronal death through perforin- and granzyme-mediated pathways, exacerbating tissue injury [15,17,35,39,40]. Although adaptive immunity intensifies the inflammatory response initially, it also plays a role in post-stroke immunosuppression, a clinically recognized phenomenon characterized by lymphocyte depletion and increased vulnerability to infections.

Acute ischemic stroke also triggers the release of DAMPs like ATP and HMGB1, which activate inflammasomes such as NLRP3 in microglia via P2X7 receptors. In response, activated microglia secrete pro-inflammatory cytokines including IL-1β and TNF-α, which stimulate endothelial cells of the cerebral vasculature to upregulate adhesion molecules (ICAM-1, VCAM-1) and release chemokines like IL-8 [17,22,33]. This signaling cascade promotes neutrophil adhesion to the endothelium and their extravasation into the brain parenchyma. Once inside, neutrophils release ROS, elastases, and proteolytic enzymes such as MMP-9, leading to degradation of the extracellular matrix and disruption of the BBB [26,35]. Monocytes are subsequently recruited through chemokines such as CCL2 (MCP-1), secreted by activated astrocytes. These monocytes differentiate into macrophages that participate in both propagation of inflammation and phagocytic clearance of necrotic debris [27].

Astrocytes also undergo reactive gliosis, releasing IL-6 and various chemokines that further amplify immune cell infiltration during the acute phase. However, in later stages, astrocytes contribute to scar formation and help restore tissue integrity by secreting structural matrix proteins and regulatory cytokines [11,41]. In the subacute phase (approximately 2–3 days post-stroke), T lymphocytes infiltrate the ischemic brain. CD4^+^ Th (T helper) cells differentiate into Th1 and Th17 subsets, producing interferon-gamma (IFN-γ) and IL-17, respectively, which sustain local inflammation and modulate innate immune cells. CD8^+^ cytotoxic T cells can directly induce neuronal apoptosis via perforin- and granzyme-mediated pathways, exacerbating tissue injury [18,32,42].

**Figure 3:**
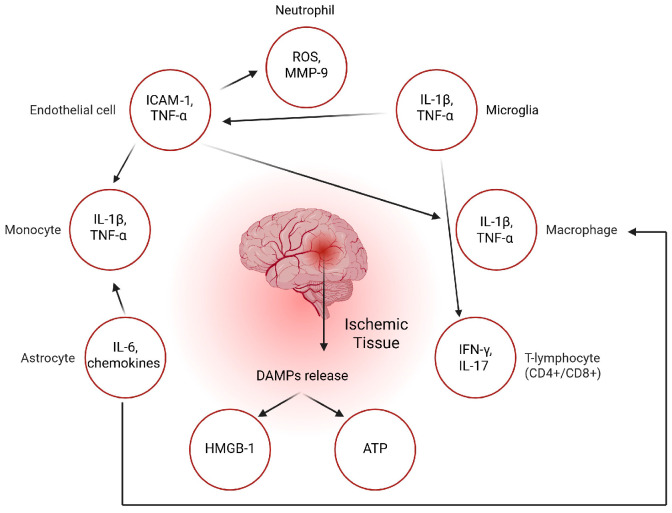
Key immune cells and their interactions in the post-ischemic brain. DAMPs (ATP, HMGB1) released by the infarcted brain tissue, act on microglia, monocytes, and astrocytes, triggering cytokine and chemokine release. The inflammatory loop sustains a damaging cycle of cytokine release (e.g., IL-1β, TNF-α, IL-6), oxidative stress (ROS), and proteolytic activity (MMP-9), further injuring the brain parenchyma. Figure abbreviations: DAMPs (damage-associated molecular patterns), HMGB1 (High-Mobility Group Box 1), ATP (Adenosine Triphosphate), IL-1β (interleukin-1 beta), TNF-α (tumor necrosis factor-alpha), ICAM-1 (intercellular adhesion molecule 1), IL-8 (interleukin-8), ROS (reactive oxygen species), MMP-9 (matrix metalloproteinase-9), IL-1 (interleukin-1), IL-6 (interleukin-6), T-lymphocytes (CD4+ helper and CD8+ cytotoxic T cells), IFN-γ (interferon-gamma), IL-17 (interleukin-17).

This intricate sequence of events, featuring crosstalk among microglia, astrocytes, neutrophils, macrophages, and lymphocytes, governs the extent of neuroinflammation, BBB compromise, and ultimately neuronal survival in the ischemic penumbra. While early and excessive inflammation promotes irreversible damage, controlled immune activation in the later stages is essential for clearing debris, modulating angiogenesis, and guiding neural repair [2,23,24,33]. The timing, magnitude, and cellular context of immune activation are therefore critical determinants of stroke progression and recovery. Specific biomarkers that reflect the evolving phases of immune activity, such as IL-6, IL-10, IL-17, MMP-9, and the NLR, are being increasingly recognized for their prognostic value and therapeutic relevance in clinical practice and trials [21,29,30,43]. Interactions between key immune cells in the post-ischemic brain are illustrated in Figure 3.

In resource-limited settings, the feasibility of implementing cytokine assays remains a major constraint. Quantification of inflammatory cytokines such as IL-6, IL-1β, TNF-α, and IL-10 typically requires ELISA-based platforms or multiplex immunoassays, which are technically demanding, costly, and not widely available outside tertiary care or research centers. These assays also suffer from longer turnaround times and require standardized sample handling, further limiting their use in acute stroke triage or bedside decision-making. In contrast, composite hematological indices such as the NLR, PLR, and SII are readily derived from routine complete blood count (CBC) panels, making them more accessible, reproducible, and cost-effective alternatives in low-resource environments. These indices offer indirect yet clinically meaningful reflections of the inflammatory response and have been validated in multiple studies as predictors of stroke severity, early deterioration, and 90-day outcomes. As such, the NLR and related indices may serve as practical surrogate markers for cytokine activity when direct measurements are not feasible. Their integration into initial stroke assessments can support early risk stratification and guide the intensity of monitoring or rehabilitation planning, particularly in health systems with limited laboratory infrastructure.

## 3. Key Inflammatory Biomarkers in Ischemic Stroke

### 3.1. Interleukin-1β (IL-1β)

IL-1β is one of the most pivotal pro-inflammatory cytokines involved in the acute phase of ischemic stroke. It is rapidly produced by activated microglia and infiltrating macrophages in response to necrotic cell debris and DAMPs. IL-1β contributes significantly to brain-blood barrier (BBB) disruption, leukocyte adhesion, and infiltration by upregulating adhesion molecules on endothelial cells and activating astrocytes and microglia. Experimental studies in mice lacking IL-1α and IL-1β showed they exhibit smaller infarct volumes after middle cerebral artery occlusion, underscoring the cytokine’s neurotoxic role in stroke pathophysiology [6,16,22].

Conversely, therapeutic blockade of IL-1 has demonstrated neuroprotective effects in preclinical models. Administration of IL-1Ra reduces infarct size, suppresses microglial activation, and attenuates neutrophil infiltration. IL-1β’s pro-inflammatory loop amplifies local damage by inducing IL-6 and TNF-α expression, forming a positive-feedback circuit of neuroinflammation. Clinically, elevated plasma IL-1β levels within the first 24 h after stroke correlate with stroke severity, larger infarct volumes, and poorer neurological outcomes. Genetic polymorphisms in IL1RN (the gene encoding IL-1Ra) have also been associated with increased stroke susceptibility, although findings have varied across populations [16,44,45].

Therapeutically, recombinant human IL-1Ra (anakinra) has been tested in clinical settings. Phase II studies demonstrated that anakinra administered within 6 h of stroke onset was safe and led to reductions in circulating neutrophils, CRP, and IL-6 levels, suggesting systemic anti-inflammatory effects. The subsequent SCIL-STROKE trial using subcutaneous IL-1Ra confirmed downregulation of inflammatory biomarkers and indicated possible improvements in functional outcomes, although larger trials are needed for validation. Despite the absence of phase III efficacy data, IL-1β remains a strong candidate for targeted intervention and a valuable biomarker reflecting acute neuroinflammatory activation after stroke [22,46].

### 3.2. Tumor Necrosis Factor-α (TNF-α)

TNF-α is a pivotal early mediator of inflammation following ischemic stroke. It is rapidly released by activated microglia and further amplified as circulating immune cells infiltrate the ischemic brain tissue [23,33,37]. TNF-α exerts diverse effects, including induction of neuronal apoptosis via TNF receptor 1 (TNFR1) signaling, generation of oxidative stress, and disruption of the brain-blood barrier (BBB) through upregulation of endothelial adhesion molecules and MMP-9. While these actions contribute to infarct expansion and edema, TNF-α also exhibits context-dependent neuroprotective functions. For example, it participates in synaptic plasticity, neurogenesis, and cell survival via TNF receptor 2 (TNFR2)-mediated pathways, particularly during the recovery phase. This dual nature has been confirmed in preclinical studies showing that TNF-α inhibition administered acutely may reduce infarct volume and neurological deficits, while delayed blockade can impair recovery by suppressing reparative processes [9,23].

In clinical settings, elevated TNF-α levels in the acute phase have been associated with larger infarcts and worse functional outcomes, although results are not entirely consistent. Some studies highlight that high TNF-α levels independently predict poor prognosis, while others suggest the association weakens after adjusting for confounders. Circulating levels of soluble TNF receptors (sTNFR1 and sTNFR2) have emerged as more stable markers, with elevated sTNFR levels linked to recurrent strokes and early post-stroke seizures. Chronic elevation of TNF-α has also been implicated in stroke risk, particularly in individuals with pro-inflammatory conditions such as diabetes or autoimmune disease. Polymorphisms in the TNF gene and persistently elevated TNF-α are associated with increased susceptibility to ischemic stroke in multiple populations [23,47,48].

Despite its established pathogenic role, TNF-α has not become a clinical target in stroke therapy. Trials involving systemic TNF inhibition (e.g., with etanercept or infliximab) have not shown benefits and may carry risk. Observational data suggest that patients with autoimmune disease on anti-TNF agents might even experience a slight increase in cerebrovascular events, possibly due to impaired vascular repair mechanisms or thrombogenic side effects [1,2,8]. Nevertheless, ongoing research is investigating more selective approaches to modulate TNF-α signaling. For example, targeting TNFR1-mediated necroptosis in cerebral endothelial cells—while preserving TNFR2-dependent protective functions—could offer a refined therapeutic strategy that mitigates inflammation without hindering post-stroke recovery. TNF-α thus remains a critical biomarker reflecting the magnitude of acute inflammation, with complex therapeutic implications [9,23].

### 3.3. Interleukin-6 (IL-6)

IL-6 is a multifunctional cytokine with a central role in the acute-phase response to ischemic stroke. It is rapidly upregulated in both brain tissue and systemic circulation within hours of vessel occlusion, and its levels correlate with infarct size, stroke severity, and prognosis [49,50]. IL-6 signals through two major pathways: classical signaling via membrane-bound IL-6 receptors (which may be regenerative or anti-inflammatory) and trans-signaling via soluble IL-6 receptors, which predominantly promote inflammation. In the early phase of stroke, IL-6 is induced by IL-1β and TNF-α and amplifies the inflammatory cascade. It contributes to leukocyte recruitment, upregulation of endothelial adhesion molecules, and stimulation of hepatic acute-phase responses, including CRP synthesis [50]. However, IL-6 also plays a role in tissue repair during later phases by supporting neurogenesis, astrogliosis, and angiogenesis—highlighting its dual role in injury and recovery [51].

Clinically, IL-6 is among the most studied biomarkers in ischemic stroke. Elevated serum levels within the first 24–48 h post-onset are associated with larger infarcts, higher NIHSS (National Institutes of Health Stroke Scale) scores, and poorer functional outcomes on the modified Rankin Scale (mRS) at 3 months [14,51,52]. Persistently elevated IL-6 levels beyond the acute phase may also predict stroke recurrence and long-term disability. Due to these associations, IL-6 is being explored as a therapeutic target. Tocilizumab, an anti-IL-6 receptor monoclonal antibody, has shown promise in preclinical stroke models, and the ongoing IRIS trial is currently evaluating its utility in patients undergoing mechanical thrombectomy. High IL-6 levels may help identify patients at risk of extensive injury who could benefit from targeted anti-inflammatory strategies [48,51,53,54].

### 3.4. C-Reactive Protein (CRP)

CRP is a well-known acute-phase reactant produced in the liver under the stimulation of IL-6 and widely used in clinical practice as a systemic inflammatory marker. In ischemic stroke, CRP levels rise within 6–24 h post-onset, often peaking between 48 and 72 h. High-sensitivity CRP (hs-CRP) assays facilitate the detection of subtle elevations that are clinically meaningful [31]. Meta-analyses and large cohort studies have confirmed that elevated admission CRP is associated with increased risk of early neurological deterioration, mortality, and worse functional outcomes at 3 months. Indeed, patients with CRP levels in the top quartile upon admission have roughly double the risk of 30-day mortality compared to those in the lowest quartile and are also at higher risk for recurrent vascular events [55,56,57].

While CRP may have a direct pathophysiological role in endothelial dysfunction and thrombosis, it is also a reliable surrogate marker for the overall inflammatory burden. CRP levels reflect infarct size, systemic immune activation, and infection status, all of which influence prognosis [31]. Persistently elevated CRP during hospitalization (for example, after thrombolysis) has been linked to poor recovery, even in patients with initially mild strokes. From a preventive standpoint, chronically elevated CRP is a risk factor for ischemic stroke, analogous to its role in coronary artery disease. Therapies such as high-dose statins, anti-IL-1β agents, and colchicine may reduce CRP and improve outcomes in high-risk populations. As such, CRP remains an accessible, cost-effective, and integrative biomarker in stroke care [26,31].

### 3.5. Interleukin-10 (IL-10)

IL-10 is a potent anti-inflammatory cytokine secreted by monocytes, macrophages, and regulatory T cells in response to tissue injury. In stroke, IL-10 modulates the immune response by inhibiting the synthesis of pro-inflammatory cytokines (e.g., IL-1β, IL-6, TNF-α), suppressing antigen presentation, and downregulating adhesion molecules on endothelial cells. It promotes neuronal survival by preventing apoptosis and limiting oxidative damage. Experimental studies have shown that IL-10 overexpression or exogenous administration reduces infarct volumes and improves neurological outcomes in animal models of ischemic stroke. However, its clinical significance is complex and time dependent [47,58,59]. A low IL-10 response in the acute phase (<24 h) is associated with unopposed inflammation, hemorrhagic transformation, and early neurological deterioration. Conversely, elevated IL-10 levels in the subacute phase (days 2–7) may indicate stroke-induced immunosuppression (SIS), increasing the risk of infections such as pneumonia and urinary tract infections [11,21,30].

This duality underscores the importance of context in interpreting IL-10 levels. While protective in moderation, excessive IL-10 may suppress systemic immunity and contribute to adverse outcomes. Some studies propose that IL-10 or IL-10/IL-6 ratios could serve as biomarkers to predict post-stroke complications, including infections or hemorrhagic conversion [6,26]. Although IL-10 is not currently a direct therapeutic target due to concerns about immunosuppression, strategies to modulate endogenous IL-10 or deliver it locally to the brain are being investigated. Ultimately, IL-10 serves as both a marker of immune balance and a potential indicator of whether inflammation is being adequately regulated in the aftermath of stroke [30,39,40].

## 4. Other Important Inflammatory Biomarkers

In addition to major cytokines like IL-1β, IL-6, TNF-α, and IL-10, several other inflammatory mediators play significant roles in ischemic stroke. Two that have received increasing attention are IL-8 and IL-17, representing innate and adaptive immune responses, respectively [22,32].

### 4.1. Interleukin-8 (CXCL8)

CXCL8 is a neutrophil-attracting chemokine secreted by activated microglia, endothelial cells, and astrocytes in response to ischemia. Blood IL-8 levels rise within hours after stroke onset and correlate with neutrophil infiltration into the brain. Elevated IL-8 levels have been linked to larger infarcts, greater early neurological deterioration, and poor functional outcomes [6]. Mechanistically, IL-8 promotes neutrophil adhesion to endothelium and entry into the brain, where these cells release proteases and reactive oxygen species, worsening brain-blood barrier (BBB) disruption. Experimental models show that blocking IL-8 signaling or deleting IL-8 receptors reduces infarct volume and neutrophil infiltration. Moreover, IL-8 may contribute to angiogenesis via stimulation of VEGF, implying a dual role in both injury and repair [42,48,60].

### 4.2. Interleukin-17 (IL-17)

IL-17, predominantly produced by Th17 cells and γδ T cells, rises slightly later, typically 24–72 h post-stroke, as part of the adaptive immune response. IL-17 amplifies inflammation by stimulating the release of IL-1β, TNF-α, and chemokines that recruit neutrophils [20,42,61]. It also directly impairs endothelial function and contributes to BBB disruption. Elevated IL-17 in plasma or cerebrospinal fluid (CSF) has been observed in patients with more severe strokes, and preclinical studies demonstrate that blocking IL-17 or interleukin-23 (IL-23) reduces infarct size and improves outcomes [6,30].

### 4.3. Other Relevant Biomarkers

Interleukin-18 (IL-18) and interleukin-12 (IL-12) are pro-inflammatory cytokines whose levels are elevated after stroke and are associated with worse clinical outcomes due to their role in enhancing Th1 responses and innate immune activation.

IL-4 and IL-13 promote M2 macrophage polarization and may facilitate repair by resolving inflammation and supporting tissue remodeling.

TGF-β and endogenous IL-1 receptor antagonist (IL-1Ra) act as regulatory cytokines in later stages, limiting immune activation and supporting recovery [6,30,62].

TREM-1 (Triggering Receptor Expressed on Myeloid Cells-1) and TREM-2 (Triggering Receptor Expressed on Myeloid Cells-2), as members of the TREM family, have been increasingly recognized for their roles in the inflammatory response and prognostic evaluation of ischemic stroke. TREM-1, found predominantly on neutrophils and monocytes/macrophages, amplifies the inflammatory response by synergizing with toll-like receptors (TLRs), leading to enhanced secretion of pro-inflammatory cytokines such as TNF-α, IL-1β, and IL-6. Elevated soluble TREM-1 (sTREM-1) levels in plasma are associated with larger infarct volumes, higher NIHSS scores, increased risk of hemorrhagic transformation, and poor functional outcomes (e.g., higher mRS scores at 90 days).

TREM-2, expressed mainly on microglia in the central nervous system, regulates microglial activation, phagocytosis, and resolution of inflammation. Upregulation of TREM-2 post-stroke is associated with enhanced clearance of cellular debris, promotion of neurorepair, and attenuation of excessive inflammation. Higher TREM-2 expression in brain tissue or cerebrospinal fluid (CSF) correlates with smaller infarct size, improved neurological recovery, and lower mortality rates.

Table 1 illustrates the main pro- and anti-inflammatory molecules involved in the pathophysiology of ischemic strokes, their main functions, timing, and clinical and prognostic implications.

**Figure 4:**
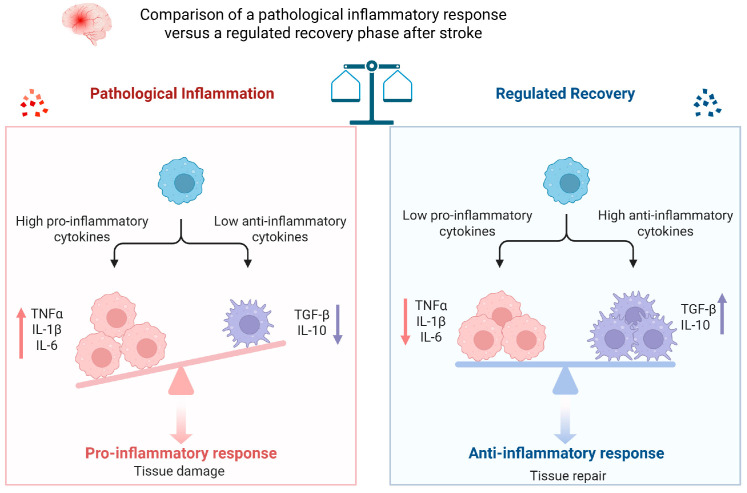
Comparison of a pathological inflammatory response versus a regulated recovery phase after stroke. In the pathological inflammation scenario *(***left***)*, there is an excessive production of pro-inflammatory cytokines (IL-1β, IL-6, TNF-α) and insufficient anti-inflammatory activity (low IL-10, TGF-β). This imbalance leads to uncontrolled inflammation and exacerbated tissue injury. By contrast, in the regulated recovery phase (**right**), anti-inflammatory mediators (IL-10, TGF-β) are prevalent and help keep pro-inflammatory cytokines in check, promoting resolution of inflammation and tissue repair. Figure abbreviations: IL-10 (interleukin-10), TGF-β (transforming growth factor-beta), IL-1β (interleukin-1 beta), IL-6 (interleukin-6), TNF-α (tumor necrosis factor-alpha).

In summary, IL-8, IL-17, and related markers broaden our understanding of the inflammatory response in stroke. Their temporal dynamics—early for IL-8, delayed for IL-17—reflect distinct phases of immune activation. Together with IL-1β, IL-6, and TNF-α, these molecules contribute to acute injury, while IL-10, TGF-β, and IL-1Ra offer counter-regulatory effects, as shown in Figure 4. Accurate profiling of these mediators may enhance prognostication and guide immunomodulatory therapies [6,9,32].

Several non-cytokine markers also reflect inflammatory burden and vascular injury. These include soluble ICAM-1, VCAM-1, E-selectin, and MMP-9. Among them, MMP-9 is particularly important for its association with hemorrhagic transformation and BBB degradation due to its proteolytic activity on extracellular matrix proteins [26,37,39,59].

## 5. Prognostic Value of Immune Biomarkers in Stroke

Inflammation-related biomarkers offer significant prognostic value beyond traditional clinical predictors in ischemic stroke. Elevated levels of pro-inflammatory cytokines, particularly IL-6, IL-1β, TNF-α, and IL-8, are consistently associated with worse neurological outcomes [1,2,3]. For example, increased IL-6 and TNF-α levels during the acute phase correlate with larger infarct volumes and higher disability scores at three months post-stroke. IL-6 levels measured upon admission have been shown to significantly correlate with both initial stroke severity and long-term functional status. Similarly, higher early concentrations of TNF-α and IL-1β are predictive of more severe neurological deficits and poorer outcomes. These cytokines thus serve as early warning markers of a more aggressive and damaging neuroinflammatory response [7,9].

Among circulating inflammatory markers, C-reactive protein (CRP) has emerged as a particularly robust prognostic biomarker due to its stability, reproducibility, and clinical accessibility. Elevated CRP levels during the subacute phase have been consistently linked with increased mortality, risk of stroke recurrence, and poor functional recovery [31]. Meta-analyses have shown that patients with CRP levels in the top quartile upon admission have approximately double the risk of 30-day mortality compared to those in the lowest quartile and are also at higher risk for recurrent vascular events. As a result, CRP is increasingly used in clinical settings for early risk stratification. A markedly elevated CRP may also prompt clinicians to consider more aggressive secondary prevention or to closely monitor for post-stroke complications such as infection [48,55].

On the other hand, anti-inflammatory cytokines such as IL-10 provide a more nuanced prognostic picture. A blunted IL-10 response in the acute phase suggests unopposed inflammation and has been associated with hemorrhagic transformation and early neurological deterioration [6]. Conversely, excessive IL-10 elevation may indicate compensatory immunosuppression and predispose patients to infectious complications such as pneumonia or urinary tract infections [4,21]. IL-10 thus serves as a dual-purpose biomarker: insufficient levels may signal heightened injury risk, while elevated levels may forecast immune suppression and secondary complications.

Beyond individual cytokines, composite inflammatory indices derived from routine blood counts have gained traction as prognostic tools. Indices such as the NLR, PLR, and SII provide integrative markers of systemic immune activation and stress [63,64]. These values rise when neutrophils and platelets are elevated and lymphocyte counts are suppressed, patterns typical of acute inflammation and stress-induced immunosuppression. Clinical studies have demonstrated that higher NLR, PLR, and SII levels are independently associated with worse outcomes after stroke [65,66]. In a 2024 cohort study involving over 500 patients, six hematological inflammatory indices, including the NLR, PLR, SII, and systemic inflammation response index (SIRI), were all linked to poor 30-day prognosis, with multivariate models identifying the NLR, PLR, and SIRI as independent predictors of early functional decline [14,67,68]. These indices are particularly appealing due to their low cost, ease of use, and ability to reflect cytokine dynamics, e.g., a high NLR may indirectly indicate IL-8- and TNF-driven neutrophilia along with cortisol-mediated lymphopenia [15,69].

In summary, immune and inflammatory biomarkers offer valuable prognostic insights following ischemic stroke. Elevated pro-inflammatory cytokines (IL-6, IL-1β, TNF-α) are associated with worse clinical trajectories, while the presence of regulatory cytokines like IL-10 and integrative markers such as CRP provide additional layers of prognostic clarity [9,70,71]. Incorporating these biomarkers into clinical prediction models, alongside established factors such as age and NIHSS score, is an active area of research, with the potential to identify high-risk patients who may benefit from closer monitoring, early intervention, or enrolment in trials of targeted immunomodulatory therapies.

## 6. Therapeutic Modulation of Inflammation: Implications for Treatment and Prevention

The central role of inflammation in stroke pathophysiology raises the possibility that immunomodulation could improve outcomes or reduce the risk of recurrence. This strategy has gained traction particularly considering repeated failures of traditional neuroprotective agents targeting excitotoxicity. Several inflammatory mediators, especially IL-1β, IL-6, and TNF-α, are now under investigation as therapeutic targets [8,22,23].

### 6.1. IL-1 Targeting

One of the most advanced approaches involves targeting the IL-1 axis. Given IL-1β’s established role in acute neuroinflammation and brain-blood barrier (BBB) disruption, pharmacologic blockade using IL-1 receptor antagonist (IL-1Ra, anakinra) has shown promise in clinical trials [5,22,24]. Phase II studies demonstrated that IL-1Ra administration in acute ischemic stroke was biologically active, reduced inflammatory cytokine levels, and was well tolerated, though sample sizes were insufficient to confirm functional benefit. Further trials are warranted, especially in subgroups with elevated inflammatory markers. Moreover, IL-1 blockade may help limit secondary injury such as hemorrhagic transformation and reperfusion damage, particularly if administered peri-thrombectomy [16]. Ongoing investigations are also exploring IL-1β-neutralizing antibodies (e.g., canakinumab) for potential neuroprotection and secondary prevention [38,61].

### 6.2. IL-6 Inhibition

IL-6 signaling is another compelling target. The IRIS trial is currently evaluating tocilizumab, an IL-6 receptor (IL-6R) blocker, as an adjunct to reperfusion therapy. The rationale stems from evidence that IL-6 mediates reperfusion injury even after successful mechanical thrombectomy, contributing to infarct expansion. Tocilizumab, already in use for cytokine release syndromes, may mitigate this response when administered acutely. However, careful monitoring is essential due to potential side effects, including transient immunosuppression and hepatotoxicity [52,53,54].

### 6.3. TNF-α Modulation

Efforts to inhibit TNF-α have produced more ambiguous results. While etanercept (a soluble TNF decoy receptor) has shown benefit in animal models, reducing infarct size and inflammation, it has not yet demonstrated efficacy in clinical trials. Concerns also persist that long-term TNF suppression could increase vascular risk. Consequently, interest has shifted to downstream pathways such as TNF-induced necroptosis, which may be selectively targeted to prevent endothelial injury without broadly impairing TNF function [9].

Findings from cardiovascular trials provide a strong rationale for inflammation-targeted stroke prevention. The CANTOS trial in post-myocardial infarction patients demonstrated that canakinumab (an IL-1β-neutralizing antibody) reduced major vascular events, including stroke, independent of lipid levels. These results support the concept that anti-inflammatory therapy alone can reduce vascular risk. While canakinumab remains costly and is associated with neutropenia, other more accessible agents are being investigated. Low-dose colchicine, which suppresses NLRP3 inflammasome activation and neutrophil recruitment, has reduced cardiovascular events in coronary disease and is currently being tested in patients with TIAs (transient ischemic attacks) or minor stroke [6,17,22]. Its actions include inhibition of IL-1β production and downstream inflammatory signaling, making it a promising candidate for both primary and secondary prevention.

Beyond pro-inflammatory suppression, attention has also turned to post-stroke immunosuppression, a major contributor to infectious complications such as pneumonia. Trials using prophylactic antibiotics like moxifloxacin have had mixed results, though targeted approaches in patients with evidence of immune exhaustion (e.g., high IL-10 levels or lymphopenia) may prove more effective. An alternative strategy involves using granulocyte colony-stimulating factor (G-CSF) to boost immune cell production. G-CSF not only promotes leukocyte recovery but also has neurotrophic properties; however, clinical trials in stroke have so far shown variable results, and its role in routine practice remains unclear [11,30,37].

Ultimately, a multi-faceted approach to immunomodulation may be necessary. Combining anti-inflammatory therapies (to limit acute injury) with strategies that prevent or reverse post-stroke immunosuppression could yield the best outcomes [60,72]. Personalized medicine approaches, such as tailoring treatments based on a patient’s inflammatory profile or genetic background (e.g., IL-1 or IL-6 polymorphisms), are on the horizon. As our understanding of the immune mechanisms in stroke deepens, it opens the door to innovative therapies that complement existing reperfusion and neuroprotective strategies.

## 7. Discussion and Future Directions

Over the past decade, mounting evidence has firmly established that immunological and inflammatory biomarkers are integral to predicting and understanding outcomes after ischemic stroke. These biomarkers not only reflect the intensity and duration of the immune response but also offer valuable insight into the potential for injury, recovery, and complications [20,60]. As stroke care increasingly moves toward precision medicine, several future directions are emerging for integrating immune biomarkers into both clinical practice and research frameworks.

### 7.1. Personalized Inflammatory Profiling

Rather than relying on isolated cytokine measurements, future studies will likely employ multi-biomarker panels, encompassing cytokines, chemokines, acute-phase proteins (e.g., CRP), and leukocyte-derived indices (e.g., NLR, PLR), to construct patient-specific inflammatory signatures. These immune profiles, analyzed using machine learning or clustering algorithms, could stratify patients by prognosis or therapeutic responsiveness [14,20,31]. Large-scale initiatives and biobanks are already collecting longitudinal blood samples to correlate inflammatory dynamics with functional outcomes and infarct evolution.

### 7.2. Real-Time and Point-of-Care Inflammation Monitoring

Technological advances in rapid bioassay platforms may soon enable bedside monitoring of key cytokines such as IL-6 or IL-1β, providing real-time assessment of inflammatory status [22,49]. This would support dynamic treatment decisions, such as the timing of intensive care de-escalation or early rehabilitation initiation. Additionally, serial inflammatory tracking could help identify patients at risk for delayed deterioration, hemorrhagic transformation, or post-stroke infection.

Despite the growing interest in using inflammatory biomarkers to guide real-time clinical decisions, several technical and logistical barriers hinder their routine application. First, assay sensitivity and specificity remain significant limitations. Many cytokines, such as IL-6 or IL-10, circulate at low picogram-per-milliliter concentrations, requiring high-sensitivity immunoassays (e.g., ultrasensitive ELISA or multiplex bead-based platforms) that are not widely available or standardized across institutions. Second, the turnaround time for conventional cytokine measurements is often several hours to days, rendering them impractical for acute stroke management where decisions must be made within narrow therapeutic windows. This is compounded by the need for specialized laboratory personnel, strict pre-analytical conditions, and sample batching, all of which delay result reporting and reduce clinical utility. Point-of-care testing platforms for cytokines are still in early developmental stages, with few validated for emergency use. Furthermore, variability in biomarker kinetics, influenced by stroke subtype, comorbidities, age, and infection, complicates interpretation of single timepoint measurements. Until rapid, automated, and clinically validated assays become broadly accessible, the utility of inflammatory biomarkers in real-time decision-making will remain limited. Bridging this gap will require collaborative efforts in assay development, regulatory validation, and integration into electronic health systems for bedside application.

### 7.3. Biomarker-Guided Immunotherapy Trials

Encouraging results from exploratory trials support the need for larger randomized controlled studies evaluating anti-inflammatory agents in stroke. Prominent candidates include IL-1 blockers (e.g., anakinra, canakinumab), IL-6R inhibitors (e.g., tocilizumab), and NLRP3 inflammasome inhibitors [9,16,17]. Another emerging approach involves cell-based immunotherapy, such as regulatory T cell or mesenchymal stem cell infusions, aimed at promoting resolution of inflammation and neurorepair. Future trials may adopt biomarker-enrichment strategies, treating only patients with elevated CRP, IL-6, or SII levels, thereby maximizing therapeutic efficiency and minimizing unnecessary exposure.

Emerging evidence supports the concept of combining immunomodulatory therapies with neurorestorative agents to enhance recovery after ischemic stroke. One promising target is brain-derived neurotrophic factor (BDNF), which promotes neuronal survival, synaptic plasticity, and axonal regeneration. BDNF expression is often suppressed in the pro-inflammatory environment of acute stroke; early attenuation of cytokines such as IL-1β, TNF-α, and IL-6 could thus create a more permissive milieu for BDNF-mediated repair. Exogenous BDNF delivery or small molecules that upregulate endogenous BDNF (e.g., SSRIs like fluoxetine, physical activity) may enhance plasticity during the subacute and chronic phases, particularly when inflammation is effectively modulated. In parallel, stem cell-based therapies, such as those leveraging mesenchymal stem cells (MSCs) or neural progenitor cells (NPCs), have demonstrated the ability to secrete anti-inflammatory cytokines, promote angiogenesis, and facilitate neurogenesis. When administered after immunosuppression is controlled, these cells may further potentiate repair by modulating microglial activation and remodeling the ischemic microenvironment. Other agents, including erythropoietin (EPO), granulocyte colony-stimulating factor (G-CSF), and IGF-1, show both immunomodulatory and neurorestorative properties and are being evaluated in combination protocols. Optimizing the temporal sequence—suppressing deleterious inflammation early, then introducing trophic and regenerative agents during the repair phase—could maximize recovery and minimize adverse effects. Such synergistic strategies represent a promising frontier in integrated stroke therapy.

### 7.4. Synergy with Neurorestorative Interventions

Post-stroke inflammation extends beyond the acute phase and modulates repair mechanisms such as neurogenesis, synaptic remodeling, and remyelination. This opens the door for combinatorial approaches that align immunomodulation with neurorestorative therapies. For instance, early anti-IL-17 therapy could reduce lesion volume and secondary damage, while late-phase immune stimulation might enhance plasticity and functional recovery [32,73]. Temporal precision will be crucial, suppressing inflammation during the hyperacute phase and boosting repair-associated immunity during convalescence.

### 7.5. Inflammation as a Target for Primary Prevention

Growing evidence suggests that chronic low-grade inflammation contributes to stroke risk, independent of traditional vascular factors. Biomarkers such as CRP and IL-6 may soon be incorporated into risk prediction models alongside blood pressure, cholesterol, and smoking status. This raises the possibility of using anti-inflammatory agents (e.g., statins, colchicine) for primary prevention in high-risk populations [34,48,54]. However, the safety of long-term immune modulation must be carefully evaluated, as immunosuppression may predispose to infections or malignancies.

### 7.6. Translational and Clinical Challenges

Despite growing interest in immunological biomarkers, several challenges limit their routine clinical application. First, there is significant variability in measurement techniques, including differences in assay sensitivity, timing of sample collection, and processing protocols, which can produce inconsistent or non-reproducible results across studies and clinical settings. Second, confounding factors such as systemic infections, malignancies, autoimmune diseases, and concurrent medications may independently alter cytokine levels or inflammatory indices, complicating the interpretation of biomarker data in stroke patients. Third, there is a lack of standardized protocols for biomarker sampling and analysis, including no consensus on ideal timepoints post-stroke for measurement or clinically meaningful threshold values. These limitations hinder biomarker comparison across cohorts and reduce confidence in their prognostic utility. Furthermore, heterogeneity in stroke subtypes and patient comorbidities adds another layer of complexity. Future research must therefore prioritize harmonization of methodologies and account for potential confounders to enable the development of reliable, clinically translatable biomarker-guided approaches.

Although there are clear associations between inflammatory markers and outcomes, causality remains difficult to prove, and therapeutic translation faces several hurdles. Stroke is a heterogeneous condition, and not all patients with elevated inflammatory biomarkers will benefit from immunosuppression. Moreover, precision is essential: excessive suppression may impair recovery or promote infection, while inadequate modulation may be ineffective. Future trials must identify the right patient subsets, timing of intervention, and optimal targets to ensure safety and efficacy [19,58].

To facilitate clinical translation, biomarkers should be integrated into existing diagnostic and prognostic workflows in a phase-specific and context-aware manner. In the hyperacute phase (within 6–24 h), measurement of pro-inflammatory markers such as IL-6, IL-1β, TNF-α, and CRP may help identify patients at risk for early neurological deterioration, hemorrhagic transformation, or poor functional recovery. These biomarkers can be obtained at the same time as routine bloodwork upon admission and interpreted alongside neuroimaging findings (e.g., infarct size, perfusion mismatch) to enhance early risk stratification. In the subacute phase (days 2–7), monitoring IL-10 levels and the NLR, PLR, and SII may inform clinicians of the immune trajectory, thereby differentiating between effective resolution of inflammation and the onset of stroke-induced immunosuppression (SIS), which may necessitate closer infection surveillance or targeted immunostimulatory interventions. Serial tracking of selected biomarkers, particularly IL-6, CRP, and IL-10, could support decisions regarding ICU discharge, early rehabilitation planning, or inclusion in clinical trials of immunomodulatory therapy. Moreover, integrating high-risk inflammatory profiles (e.g., elevated IL-6 + high NLR) into electronic health records with automated alerts may enable real-time, personalized interventions. Ultimately, establishing institutional protocols for standardized biomarker sampling at defined timepoints (e.g., admission, 48 h, 7 days) and interpreting them in the context of stroke subtype, imaging, and comorbidities will be essential for effective clinical implementation.

## 8. Conclusions

Immunological and inflammatory biomarkers, including IL-1β, IL-6, TNF-α, IL-10, IL-8, IL-17, and CRP, have become indispensable tools in stroke research, offering insight into how the immune system shapes both the extent of brain injury and the trajectory of recovery [6,8,9,30]. These molecules are not merely reflections of damage but active participants in the pathophysiological cascade, and they hold considerable prognostic value. Elevated pro-inflammatory markers generally correlate with larger infarct volumes, higher rates of disability, and poorer functional outcomes, while anti-inflammatory signals such as IL-10 convey a more regulated immune response and may predict better recovery [74].

These biomarkers are paving the way for a new class of stroke therapies aimed at modulating the immune response. From IL-1 blockade to IL-6 receptor inhibition and inflammasome targeting, clinical trials are now leveraging our growing understanding of neuroinflammation to guide therapeutic strategies. In parallel, hematological indices such as the NLR, PLR, and SII are being validated as cost-effective, readily available proxies for immune activity, offering clinicians practical tools for early risk stratification and management decisions [13,61,68,69].

The interaction between the immune and nervous systems in stroke is intricate and dynamic, influenced by time, comorbidities, and genetic background. Yet, untangling these relationships offers a transformative opportunity to move toward personalized, pathophysiology-guided stroke care [36,47]. As we integrate biomarkers into clinical workflows, their use may extend from prognosis to therapy selection, rehabilitation planning, and secondary prevention.

Ultimately, by monitoring and modulating the inflammatory response, clinicians may be able to reduce secondary brain injury, enhance repair mechanisms, and prevent recurrent events. This strategy has the potential to improve survival, optimize functional recovery, and reduce the overall burden of stroke, a critical goal in the face of aging populations and rising global incidence. The next decade of research will be crucial in translating these insights into standard-of-care tools and treatments, marking a paradigm shift in how stroke is managed at both the individual and population level.

## Figures and Tables

**Table 1:** Main pro- and anti-inflammatory molecules involved in the pathophysiology of ischemic strokes.

Biomarker	Classification	Main Functions	Clinical/ Prognostic Implications	Timing of Elevation Post-Stroke	Clinical Threshold/Risk Cut-Off	References
IL-1β	Pro-inflammatory	Activates microglia; initiates inflammatory cascade	Linked to larger infarct volume and worse outcomes	Peaks within 6–24 h	>15 pg/mL may indicate severe inflammation	[4,6,8,22]
IL-6	Pro-inflammatory	Promotes acute-phase response and leukocyte recruitment	High levels predict poor functional outcome and mortality	Rises within hours; persists for days	>7 pg/mL associated with unfavorable outcome	[9,10,36]
TNF-α	Pro-inflammatory	Promotes apoptosis; disrupts the brain-blood barrier (BBB)	Correlates with infarct size and neurological decline	Peaks within 6–24 h	>20 pg/mL linked to higher mortality	[9,23,24,26]
IL-10	Anti-inflammatory	Inhibits pro-inflammatory cytokines	Protective at moderate levels; high levels are linked to infection risk	Increases within 24–72 h	>30 pg/mL may predict post-stroke infections	[6,29,30]
TGF-β	Anti-inflammatory/Regulatory	Promotes repair and modulates immunity	May enhance recovery; precise prognostic value is still uncertain	Late elevation (days to weeks)	>10 ng/mL may reflect tissue remodeling and repair	[24,27,30]
IL-17	Pro-inflammatory	Neutrophil recruitment; amplifies inflammation	Associated with worse outcome and BBB disruption	Peaks within 24–72 h	>5 pg/mL associated with infarct expansion	[23,32]
IL-8	Pro-inflammatory	Neutrophil chemotaxis and activation	High levels predict early neurological deterioration	Early peak within 24 h	>30 pg/mL linked to larger infarcts	[8,18,21,58]
MMP-9	Proteolytic enzyme	Degrades extracellular matrix; promotes BBB breakdown	Elevated levels predict hemorrhagic transformation and poor recovery	Peaks at 24–48 h	>140 ng/mL predicts hemorrhagic transformation	[26,29,33,40]
HMGB1	DAMP/Alarmin	Released by necrotic cells; promotes cytokine release via TLR4/RAGE	Correlates with infarct size, edema, and poor outcome	Peaks within 24 h; persists if infarct is large	>8 ng/mL may indicate increased risk of poor outcome	[8,30,60]
CRP	Acute-phase reactant	Non-specific marker of systemic inflammation	High levels are linked to infarct progression, mortality, and recurrent stroke	Rises within 12–24 h	>10 mg/L associated with poor outcomes	[2,3,4,31,55]
S100B	Astroglial protein	Reflects astrocytic injury and BBB disruption	High serum levels indicate brain damage severity	Peaks at 24–48 h	>0.35 μg/L associated with poor prognosis	[8,23,26]

## Data Availability

No new data were created or analyzed in this study. Data sharing is not applicable to this article.
